# Associations between implicit and explicit affective inhibitory control, trait rumination and depressive symptoms

**DOI:** 10.1192/j.eurpsy.2022.1427

**Published:** 2022-09-01

**Authors:** M. Nahum, T. Van Vleet, J. Jordan, O. Shimony, O. Bonne

**Affiliations:** 1 The Hebrew University of Jerusalem, School Of Occupational Therapy, Jerusalem, Israel; 2 Posit Science Corp., R&d, San Francisco, United States of America; 3 Dominican University of California, Department Of Psychology, San Rafael, United States of America; 4 Hebrew University of Jerusalem, School Of Ot, Jerusalem, Israel; 5 Hadassah Medical Center, Department Of Psychiatry, Jerusalem, Israel

**Keywords:** Depression, inhibition, rumination, Executive function

## Abstract

**Introduction:**

Inhibitory control is the executive function component which underlies one’s ability to maintain goal-directed behavior by inhibiting prepotent responses or ignoring irrelevant information. Recent models suggest that impaired inhibition of negative information may contribute to depressive symptoms, and that this association is mediated by rumination. However, the exact nature of this association, particularly in non-clinical samples, is unclear.

**Objectives:**

The goal of the current study was to assess the relationship between inhibitory control over emotional vs. non-emotional information, rumination and depressive symptoms.

**Methods:**

A non-clinical sample of 119 participants (mean age: 36.44 ± 11.74) with various levels of depressive symptoms completed three variations of a Go/No-Go task online; two of the task variations required either explicit or implicit processing of emotional expressions, and a third variation contained no emotional expressions (i.e., neutral condition).

**Results:**

We found that for participants who reported elevated depressive symptoms, their inhibitory control ability was reduced for all three task variations, relative to less depressed participants. However, for the task variation that required implicit emotion processing (rather than explicit), depressive symptoms were associated with inhibitory deficits for sad and neutral, but not for happy facial expressions. An exploratory analysis showed that the relationship between inhibition and depressive symptoms occurs in part through trait rumination for all three tasks, regardless of emotional content.

**Conclusions:**

Collectively, these results indicate that elevated depressive symptoms are associated with both a general inhibitory control deficit, as well as affective interference from negative emotions, with implications for the assessment and treatment of mood disorders.

**Disclosure:**

No significant relationships.

